# Resilience of Urban Transport Network-of-Networks under Intense Flood Hazards Exacerbated by Targeted Attacks

**DOI:** 10.1038/s41598-020-66049-y

**Published:** 2020-06-25

**Authors:** Nishant Yadav, Samrat Chatterjee, Auroop R. Ganguly

**Affiliations:** 1 Sustainability and Data Sciences Laboratory, Department of Civil and Environmental Engineering, Northeastern University, Boston, MA USA; 2 Computing and Analytics Division, National Security Directorate, Pacific Northwest National Laboratory, Richland, WA USA

**Keywords:** Engineering, Complex networks, Climate-change impacts

## Abstract

Natural hazards including floods can trigger catastrophic failures in interdependent urban transport network-of-networks (NoNs). Population growth has enhanced transportation demand while urbanization and climate change have intensified urban floods. However, despite the clear need to develop actionable insights for improving the resilience of critical urban lifelines, the theory and methods remain underdeveloped. Furthermore, as infrastructure systems become more intelligent, security experts point to the growing threat of targeted cyber-physical attacks during natural hazards. Here we develop a hypothesis-driven resilience framework for urban transport NoNs, which we demonstrate on the London Rail Network (LRN). We find that topological attributes designed for maximizing efficiency rather than robustness render the network more vulnerable to compound natural-targeted disruptions including cascading failures. Our results suggest that an organizing principle for post-disruption recovery may be developed with network science principles. Our findings and frameworks can generalize to urban lifelines and more generally to real-world spatial networks.

## Introduction

According to the World Economic Forum’s Global Risks Report 2019^1^, extreme weather events are the global risks of highest concern. Heavy precipitation, along with associated flooding in urban megaregions, has been on the rise both in intensity and frequency under the dual forcings of climate change and rapid urbanization. Consequently, critical urban lifeline infrastructure systems (CULIS) across the globe are under stress, with multimodal urban transport systems (MUTS) among the worst affected by urban flooding. Moreover, transportation networks are functionally interdependent with each other and on other infrastructure systems such as the power grid and communication networks. Thus, even a limited disruption in one system can spiral out of control leading to severe loss of lifeline functions. Further, as MUTS are becoming increasingly connected and autonomous, security experts have pointed to the growing threat of opportunistically targeted cyber-attacks designed to take advantage of natural hazard events^2^.

Numerous definitions of resilience have been proposed in the literature^3^, although here we adopt the most widely cited provided by the US National Academy of Sciences: *“the ability to prepare and plan for, absorb, recover from and more successfully adapt to adverse events”*^4^ . The growing threat of natural, targeted and compound disruptions on MUTS calls for an urgent need to analyze and build resilience at a system level. Compound disruptions here refer to disruptions which may occur simultaneously or sequentially where the network has not fully recovered from the initial disruption. For example, a targeted cyber-physical attack in conjunction with a natural hazard in order to exploit the already weakened network capacity. Furthermore, the multiscale and interconnected nature of MUTS, combined with the inherent unpredictability of extreme weather events, make the resilience task even more challenging^6^.

Conceptual frameworks for resilience are available in the extant literature^6–7^, but limited work has been done on modeling and quantifying MUTS resilience with the aim of generating actionable insights for stakeholders. The recently demonstrated “universality” of network science-based approaches^8–12^ provide a natural method of choice for quantifying resilience of networked systems such as the MUTS. One of the most widely studied property in network science is the robustness of a network given the failure of a subset of its nodes. Inspired by percolation theory, the giant (largest) connected component of the network is typically treated as a proxy for the state of functionality in the network^13^. This approach has helped in understanding the robustness properties of different network topologies and the corresponding systems they represent. Recent studies in this area range from robustness analysis of specific infrastructure systems such as the power grid^14^ and MUTS^11,15^, to “universal” theories of resilience^16,17^ which consider network dynamics and topology. Besides robustness, researchers have applied network science and engineering to find optimal attacker strategies^18^ as well as most effective post-failure recovery sequences^19^ in infrastructure networks.

While prior studies in transportation networks have primarily focused on single networks (and single disruptions)^11,20^, real-world infrastructure networks rarely appear in isolation. The interconnected and interdependent network-of-networks (NoNs) give rise to a rich topology, which in turn exhibits behavior that may be different from single-layer networks. A recent study^21^ presented an analytical framework to study the robustness in a system of two interdependent networks and found that such interdependence makes them vulnerable (or, less robust) compared to single networks. The subsequent literature developed generalized results for ‘n’ interdependent networks^22^, diverse failure schemes^23^, as well as what has been referred to as “universal theories” for cascading failures in single^16^ and interdependent networks-of-networks^17^.

The existing literature cited above has largely looked at idealized NoNs from a theoretical standpoint to characterize their physical properties such as percolation threshold and phase transitions. However, theoretical frameworks may not directly apply to real-world networks having topologies which are, often markedly, different. Thus, recent research in real spatial networks (e.g. MUTS)^24,25^, which have not received much attention in the network science literature, reveal that spatial constraints render them significantly more vulnerable compared to their non-embedded NoN counterparts. In addition, prior research has predominantly considered random, natural, and targeted failures individually, however as mentioned earlier, there is a growing need to study compound failure scenarios arising from natural hazards and cyber-physical attacks.

Additionally, quantifying resilience entails measuring not just the failure but also the recovery processes. Infrastructure recovery includes restoring network functionality through maximizing post-disruption network flow and/or reconstructing network connectivity while minimizing the temporal and monetary cost of restoration. Current recovery methods can be broadly classified into optimization-based^26,27,28^ and network-science based methods^19,29^ which prioritize a recovery sequence based on pre-defined metrics such as network centrality measures. Although optimization techniques including mixed-integer programming and greedy algorithms have been used to derive efficient recovery strategies, they are computationally expensive, and may even be prohibitive for large networks. For infrastructure networks such as MUTS, where real-world node importance is highly corelated with node centrality, centrality-based heuristic methods can provide comparable or even faster recovery sequences. We test this hypothesis while generating post-failure recovery sequences for LRN.

From the network-science perspective, a long-term goal may be to arrive at universal theories for resilience in spatial NoNs. However, given the complexity and diversity among network topologies and failure scenarios, as well as the size of the networks, the possibility of arriving at such universal theories may need to be examined through hypothesis-driven studies. Meanwhile, urgent solutions are needed for such networks; thus, in our opinion a first step would be hypothesis-driven research focusing on specific aspects of the overall problem. Here we address three hypotheses: 1) the spatially constrained *MUTS NoN topology renders it more vulnerable* compared to other spatially non-embedded NoN topologies; 2) a limited targeted attack in conjunction with an intense natural hazard, may cause disproportionate network failure compared to single hazards. *3) Network-centrality based recovery comparably or even outperform optimization-based methods for MUTS*.

The primary network-science contribution of this work is to present a generalized computational framework for a quantitative understanding of resilience, including robustness and recovery, of real-world and spatially constrained urban transportation NoNs. Second, we demonstrate the framework on the LRN and obtain insights that may generalize to other CULIS systems globally. Our analysis has been performed in two parts. First, we focus on understanding the inherent vulnerabilities of the network due to spatial constraints and network sparsity. Second, the London NoN is tested against a suite of failure scenarios – random, targeted attacks, and natural hazards – as well as the failures owing to compound failures. The insights derived are expected to generalize to other MUTS datasets while the caveats and open challenges may lead to new hypotheses which can be further tested on MUTS datasets globally.

## Results

The robustness of the network is measured by the rate at which functionality is lost given the failure of a subset of its nodes. Inspired from percolation theory, the giant connected component (GCC) is treated as a proxy for the instantaneous functionality of the network. At each discrete time step (during failure), the size of GCC can be calculated from the adjacency matrix, which encodes all the information about the network, using an appropriate algorithm such as Kosaraju’s depth-first search algorithm^30^.

1 $$\begin{array}{c}G(n)\, \sim f({A}_{ij}\times W(n))\end{array}$$

where, $G(n)$ is the size of the giant connected component at timestep $n$ in the failure process. ${A}_{ij}$ is the $(N\times N)$adjacency matrix and $W(n)$ represents failure operating as a matrix transformation on the adjacency matrix, updating the network information at each failure step.

For NoNs (considering a two-network case), where failure also cascades from the interdependent network, the effective failure matrix becomes:

2 $$\begin{array}{c}{W}^{{\prime} }(n)=W(n)\times {C}_{1,2}\end{array}$$

and,

3 $$\begin{array}{c}G(n)\sim f({A}_{ij}\times {W}^{{\rm{{\prime} }}}(n))\end{array}$$

where, ${C}_{1,2}\,$is the dependency matrix capturing the node dependency between the two networks. Therefore, in the case of a two-layer NoN topology, the robustness of each network $i$ can be codified as follows:

4 $$\begin{array}{c}{F}_{i}(n)=1-\frac{{G}_{i}(n)}{{G}_{i}(0)}\end{array}$$

where, $F(n)$ is the dynamic network functionality as nodes are removed from the network. ${G}_{i}(0)$ is the initial GCC size of the fully functional network.

In case of compound disruptions, when a second disruption $T(n)$ occurs after a fraction of nodes $N-{N}^{{\rm{{\prime} }}}$ are removed due to the initial failure $W(n)$. Starting from ${N}^{{\rm{{\prime} }}}$ the third term in equations^6^ and^8^ below caters to the second failure $T(n)$. ${{A}^{{\rm{{\prime} }}}}_{ij}$ and ${{C}^{{\rm{{\prime} }}}}_{1,2}$ is the updated adjacency matrix and dependency at the last instance of initial failure.

5 $$\begin{array}{c}{F}_{i}(n)=1-{\left\{\frac{{G}_{i}(n)}{{G}_{i}(0)}\right\}}_{n=0}^{N-{N}^{{\rm{{\prime} }}}}-{\left\{\frac{{G}_{i}^{{\rm{{\prime} }}}(n)}{{G}_{i}(0)}\right\}}_{n=N-{N}^{{\rm{{\prime} }}}}^{N}\end{array}$$

where,

6 $$\begin{array}{c}{{G}^{{\rm{{\prime} }}}}_{i}^{(n)}\sim f({{A}^{{\rm{{\prime} }}}}_{ij}\times T(n)\times {{C}^{{\rm{{\prime} }}}}_{1,2})\end{array}$$

### Network vulnerability

**Figure 1:**
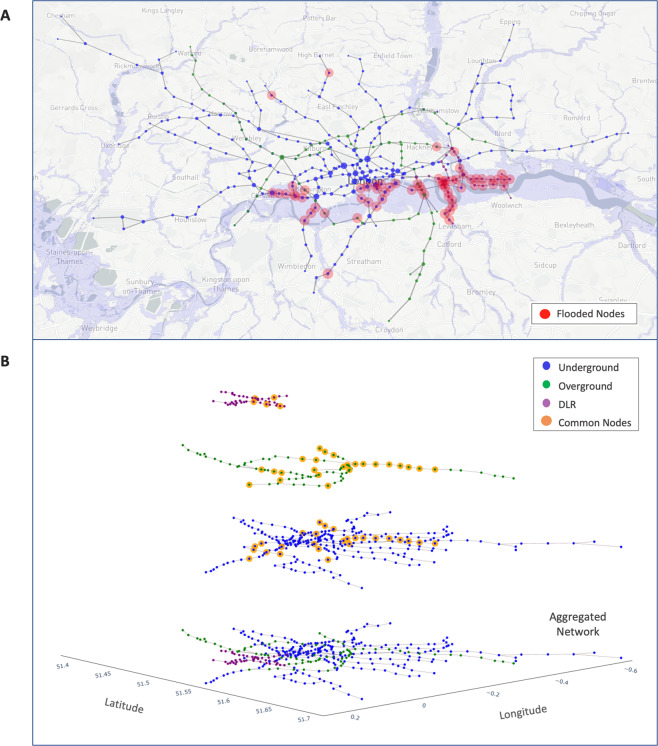
Flood on London Rail Network-of-Networks. Geocoded network over the map of London along with the 100–1000-year flood risk map (in purple) of river Thames and its tributaries. Nodes in red are the impacted nodes. **(A)** Top view - impacted nodes across all networks (total = 399, impacted = 65). (**B)** Multilayer view - impacted nodes in each network – 31 (Underground), 7 (Overground) and 27 (DLR). Nodes in orange are the shared (common) nodes across the three networks. [(**A**) Created using Mapbox -https://www.mapbox.com/].

The London Rail ‘Network-of-Networks’ (LRN) comprises of three urban rail networks – Underground, Overground, and the Dockland Light Rail (DLR) – interconnected via shared nodes (see Methods: Common Stations). Although the Underground sub-network itself comprises of 11 different lines, in this work all the Underground lines are considered as a single network. For details on nodes and links in each network, see Methods: Network Structure. Figure 1(A) shows the geocoded LRN over the map of London. Nodes in red indicate nodes that would be flooded by 100–1000-year floods on the river Thames (relevant details are discussed later).

**Figure 2:**
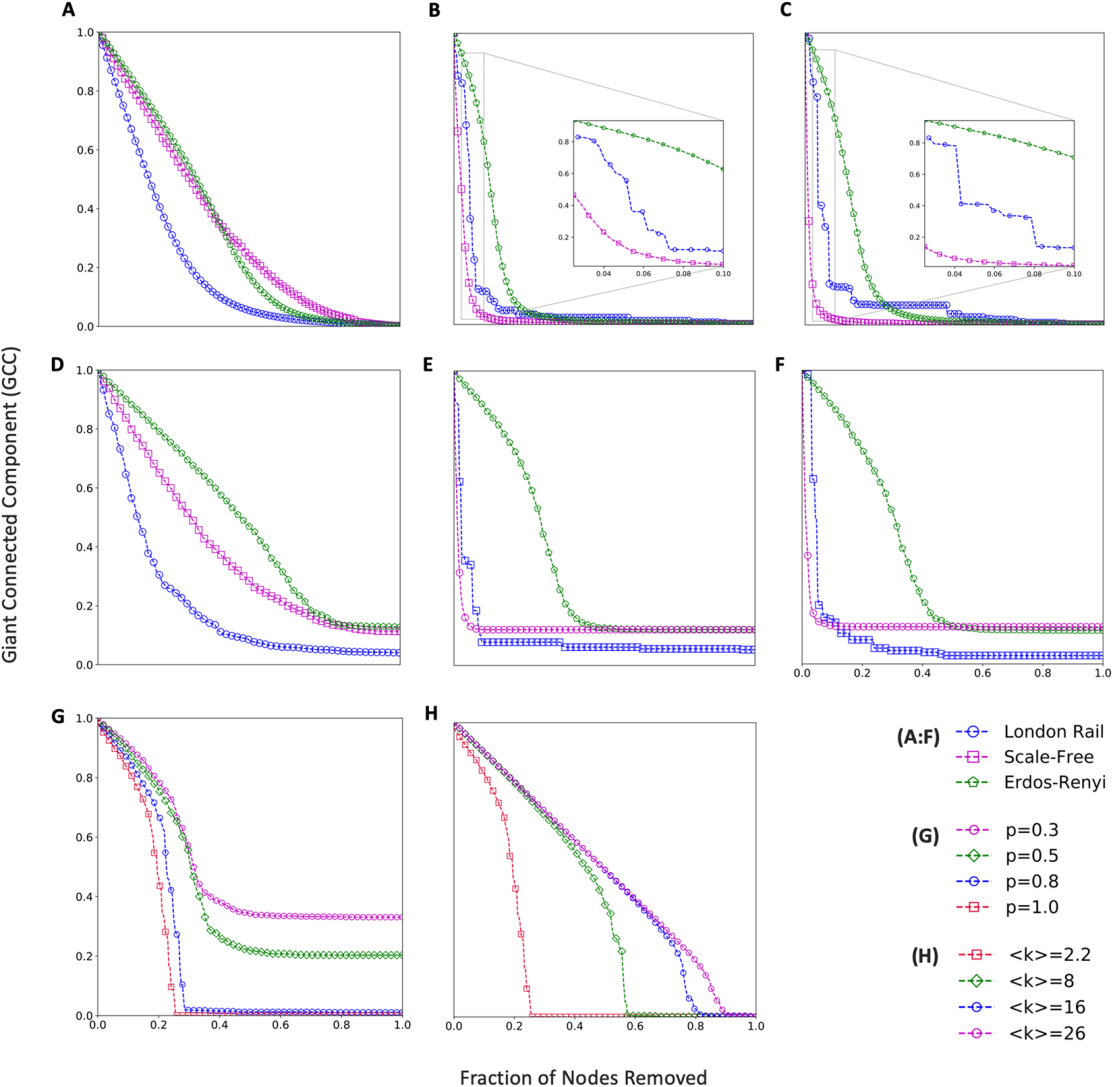
Robustness of London Rail Network. The size of the largest (giant) connected component is plotted as nodes are removed from the network. Simulated Erdos-Renyi (Random) and Scale-Free networks are used as benchmark. In figure labels (**A**–**C**) the network is treated as aggregated (single-layer) and in (**D**–**F**) as a multilayer network-of-networks (NoN). Nodes are removed randomly in (**A**) and (**D**); in a targeted manner based on degree ((**B**) and (**E**)) and betweenness ((**C**) and (**F**)). To simulate cascade failure in the NoN case **((D**–**F))**, nodes are removed only from the underground network. **(G**,**H)** Impact of interdependency (coupling strength) and average network degree, respectively, on robustness demonstrated using two coupled Erdos-Renyi networks. Each plot is an ensemble mean of 20 independent runs.

Robustness of networks, including transportation networks, have been examined in terms of the relative size of the giant (largest) connected component (GCC) when a fraction of nodes is removed^13,21^. Figure 2 compares the robustness of the London Rail network (LRN) with equivalent Erdos-Renyi (Random) and Scale-Free (SF) network representations, subjected to random and targeted failure scenarios (see Methods: Failure Scenarios). Equivalence of the real (LRN) and simulated (Random and SF) networks in this context implies that the total number of nodes, layers and average degree are kept identical. Initially, our analysis treats the inter-network links in the LRN as connectivity (enabling) links. For random failure, 20 independent runs are conducted where nodes are removed at random and the ensemble mean for the GCC is plotted. For targeted failure, two of the most common centrality measures – node degree and betweenness – are considered. Similar failures are applied to the synthetic Erdos-Renyi (ER) and Scale-Free networks and results are shown in Fig. 2 for (A) random failure, (B) targeted (degree) and (C) targeted (betweenness).

When inter-network links are treated as connectivity links, the overall network effectively behaves like a supra-single network with distinct communities. Thus, a question from the network science perspective that may arise is why represent the LRN as a NoN in the first place. Our rationale for the proposed NoN representation is that by increasing topological granularity (i.e., dividing the NoN into interconnected networks) new insights can be derived which otherwise would not be possible. Thus, insights about the *interdependence* of the different network layers and the ensuing *cascading failure* may be better developed through a NoN representation. Furthermore, this provides a general framework for interdependent critical infrastructure networks such as the power grid and communication networks. Here the LRN NoN representation allows an examination of the interdependency between the three different yet coupled rail networks (Underground, Overground, DLR).

Based on the consideration above, the next part of our analysis treats the inter-network links as dependency links, i.e., if a node fails in one layer, then its dependent node in the corresponding dependent layer fails as well. Without loss of generality, in our work, we consider the two largest layers, specifically, Underground and Overground Rail networks, and study the system by removing nodes only from the Underground layer. As Underground nodes are removed, our representation removes the dependent nodes in the Overground layer, which in turn provides a feedback effect on the Underground network. Thus, the feedback between the layers leads to a cascading failure scenario. Supplementary Fig. S7 presents a schematic diagram to illustrate this cascading failure scenario. Corresponding results for this case are shown in Fig. 2(D) for random failure, (E) targeted failure (based on degree) and (F) targeted (betweenness).

Under random failure, the LRN NoN is least robust compared to both ER-ER and SF-SF NoNs (Fig. 2(D)). Under targeted failure (Fig. 2(E,F)), the LRN NoN is significantly less robust compared to ER-ER and comparable to SF-SF NoN despite not obeying the scale-free property which renders SF networks extremely fragile under targeted attacks^31^. Even when the LRN is considered as an aggregated single-network and compared with single ER and SF network, its robustness characteristics are same as above (Fig. 2(A), random 2(B) and (C), targeted). For assortative coupling (like node dependent on like node; the case of LRN), robustness properties for interdependent ER and SF networks are well established in the literature^32^ (Table 1). Using these as a benchmark, we contrast the LRN as ‘fragile-fragile’, i.e. fragile under both random and targeted failures.

**Table 1:** Robustness of Interdependent ER and SF Networks under Random and Targeted Failure.

Network	Random Failure	Targeted Attack
ER-ER	Robust	Robust
SF-SF	Robust	Fragile

The relatively high vulnerability of real-world transportation network like the LRN can be attributed to the sparsity of connections and a narrow degree distribution concentrated around ~2 (Supplementary Fig. S8), resulting in the presence of a few highly critical nodes (junction stations). Moreover, the topology can be described as 2D lattice-like and spatially constrained^25^, i.e. nodes are linked mostly to nearby nodes as dictated by efficient design considerations (since creating each physical link has an associated cost). Consequently, while spatial networks have short links, random networks do not have a characteristic link length because nodes can be spatially mobile, and links can be across the networks thus leading to a compact topology and higher robustness.

Furthermore, the average shortest path length – a commonly used metric in network science - is significantly higher in the LRN (Table 2), which signifies that a select few nodes are extremely critical through which most of the shortest paths pass (i.e. there is an absence of hops). These are usually the transit points in transportation networks, and their failure may lead to significant loss of network functionality in quick time. On the contrary, the LRN robustness is boosted by the fact that degree correlation between dependent nodes is one (since dependent nodes are identical in this case). Indeed, it has been shown that high inter-similarity in spatial NoNs makes them more robust^33^.

**Table 2:** Average Shortest Path Length for LRN, ER and SF Network Topologies.

Network Type	Average Shortest Path Length
London Rail Network	~13
ER	~4.8
SF	~6.1

For a more comprehensive understanding, we studied the role of connectivity and dependency strength on the robustness of NoNs in general. We consider two equal sized ER random networks under random failure. Dependency ratio ‘p’ is defined as the fraction of nodes interdependent in each network. For different values of p, robustness profiles are plotted (Fig. 2(G)). We find that as dependency increases, the coupled network fails faster. However, below a certain dependency ratio p, the coupled network doesn’t fail completely, as can be seen in the plot where the final GCC size never approaches zero. For higher values of p, not only do we observe a complete failure, but also a transition from a second-order to first-order (abrupt) failure profile, which is in conformation with existing literature^34^. To understand the role of connectivity, keeping p = 1, the average degree < k > is varied in both networks (Fig. 2(H)). It is found that coupled networks with higher average degree are more robust. In other words, redundancy has a positive impact on network robustness.

### Flood failure – robustness and recovery

#### Identifying flooded nodes

The London Rail NoN is geocoded over the map of London using the lat-long coordinates of the stations (Fig. 1). Next, the 100–1000-year flood risk map on river Thames, obtained from the UK’s Environment Agency’s database^35^, is overlaid on this network. Figure 1(A) shows the impacted stations (nodes) lying in the flood risk zone - 65 stations in total are impacted.

**Figure 3:**
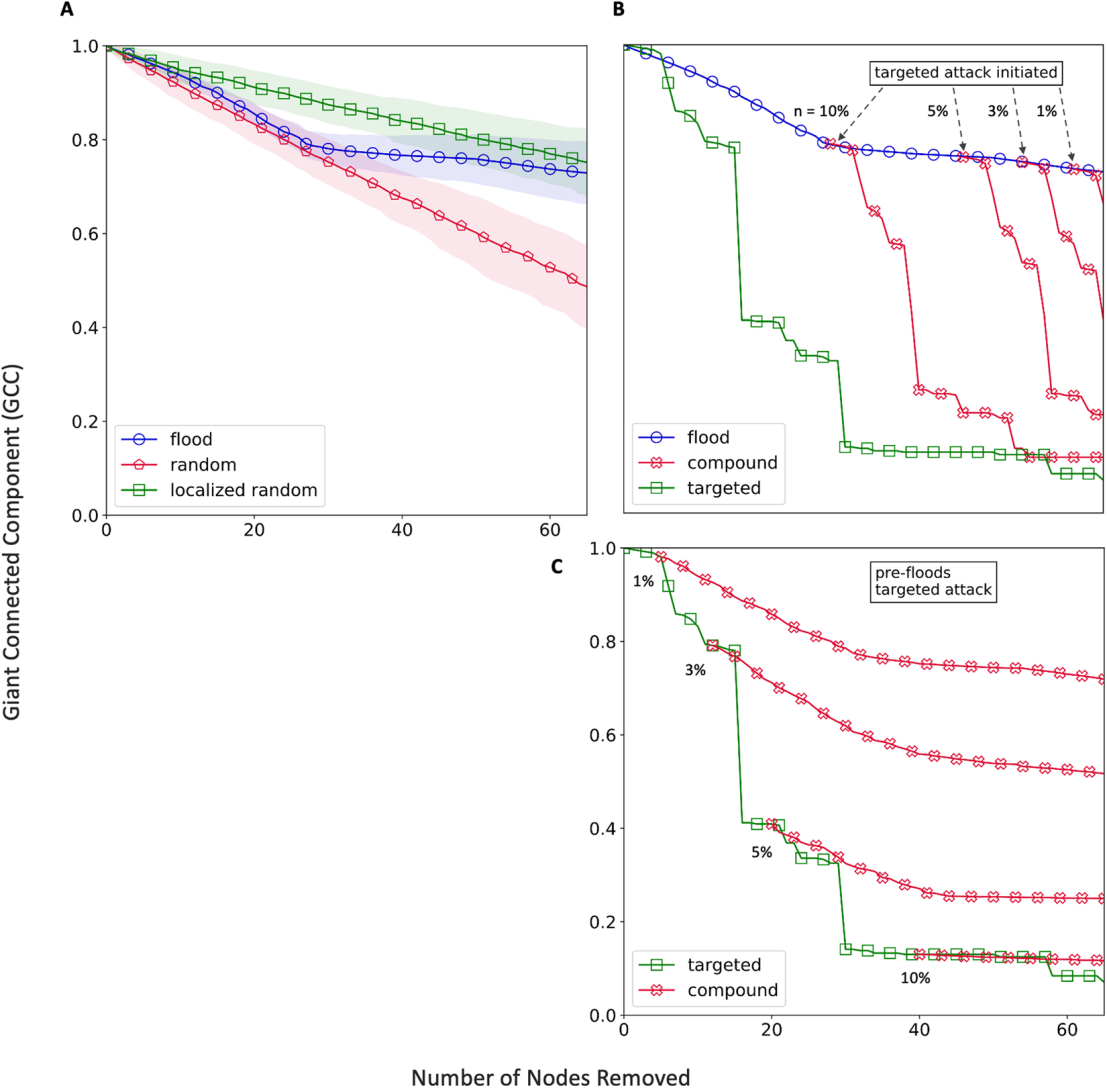
Robustness of the London Rail network under (**A**) flood failure and two types of random failure (overall and localized). (**B**) compound failure where n% nodes (of total) are removed in a targeted manner after certain nodes have failed due to flooding. (**C**) compound failure with targeted attack before flooding.

After the flooded nodes are identified, we test the LRN against different failure scenarios – flood-induced, random, local, targeted and compound. Their mechanism is described in the Methods section. Similar to before, we use the giant connected component (GCC) to quantify the robustness of the network. We find that flood induced failure has a distinct profile compared to both the types of random failures (Fig. 3(A)). Approximately 80% of the total damage happens within the first one-third node failures in the case of flooding. The kink (sharp drop in slope) in the flood-induced failure curve is indicative of the potential for sudden large-scale failure of the network as well as the existence of a critical point. The reason for sudden failure in flooding is that the average degree of the flooded nodes is higher than the average degree of the overall network (Supplementary Fig. S9). In other words, more critical or important nodes are in the flood risk zone. For instance, in the case of the London subway, the busiest stations like King’s Cross and Waterloo are next to river Thames and in the riparian zone. Historically, cities have developed around rivers and the important stations and junctions were traditionally built close to the river body to provide quick transition to water transportation. However, the same design philosophy, which may not be as valid in modern times, leaves these transportation systems highly vulnerable to flooding.

#### Compound disruptions – flood and targeted attacks

Next, we consider the potential loss of functionality due to a compound disruption, as exemplified by opportunistic targeted attacks in conjunction with a natural hazard. Specifically, two scenarios are simulated: (1) flood-failure followed by a targeted attack, and (2) a targeted attack preceding flood. To have a fair comparison the total number of failed nodes (flood + targeted) are kept equal to the case of flood-only failure. In both cases (Figure 3(B) and C, it is observed that compound disruptions cause network-wide failure. However, the major proportion of the perceived network-wide failure is contributed by the ‘limited’ targeted attacks in both scenarios. E.g., for a 5% targeted attack, ~68% of the total loss of giant connected component (GCC) is due to post-flooding targeted attacks. While the loss is 74% when targeted attacks happen first. A plausible explanation for higher GCC loss when targeted attacks occur first is that on a fresh network the adversary gets to take out the most critical nodes first (e.g. by degree or betweenness). On the other hand, when flooding occurs first, such nodes with highest criticality may fail during flooding itself but not in the most ‘optimized order’. Besides, a true compound failure impact is not observed – i.e. where failure from the compound disruptive event potentially equals or exceeds the aggregated failure from isolated targeted and natural disruptions. One caveat to this analysis is the assumption that compound failure profile can be partitioned cleanly into targeted and natural phases may not be entirely realistic. However, we do observe that when targeted attacks occur before flooding, 6% more total loss of GCC takes place indicating that compound failure sequence does impact the system differently. While the present work is an early exploration of networked systems under compound disruptions, relaxing the binary node failure condition, allowing partial functionality and exploring ingenious targeted attacks (beyond what are just based on node criticalities, such as attacks which can preempt and worsen the impact of the subsequent disruption) can provide a more granular analysis.

#### Recovery strategies

To quantify the relative performance of different recovery methods, we first define a network functionality metric that allows us to incorporate restoration cost and capacity addition per node. The weighted inverse distance has been used to measure the efficiency (or functionality) of transportation networks^37^. We modify it as follows:

$$\Gamma =\mathop{\sum }\limits_{i}^{N}\mathop{\sum }\limits_{j\ne i}^{N}\frac{{w}_{i}{w}_{j}}{{c}_{i}}\ast \frac{1}{{d}_{ij}}$$

where, $\Gamma$ is the modified weighted inverse distance of the network, ${w}_{i}$ and ${w}_{j}$ denote the weights (or traffic flow) of node $i$ and $j$. They denote the capacity addition during the recovery process. ${c}_{i}$ is the restoration cost for node $i.$ ${d}_{ij}$ is the shortest distance between all the pair of nodes $i$ and *j* restored by that point. It is infinite if nodes are not connected.

Network functionality is then defined as,

$$F=\frac{\Gamma }{{\Gamma }_{0}}$$

where, ${\Gamma }_{0}$ denote the modified weighted inverse distance of the network before failure.

**Figure 4:**
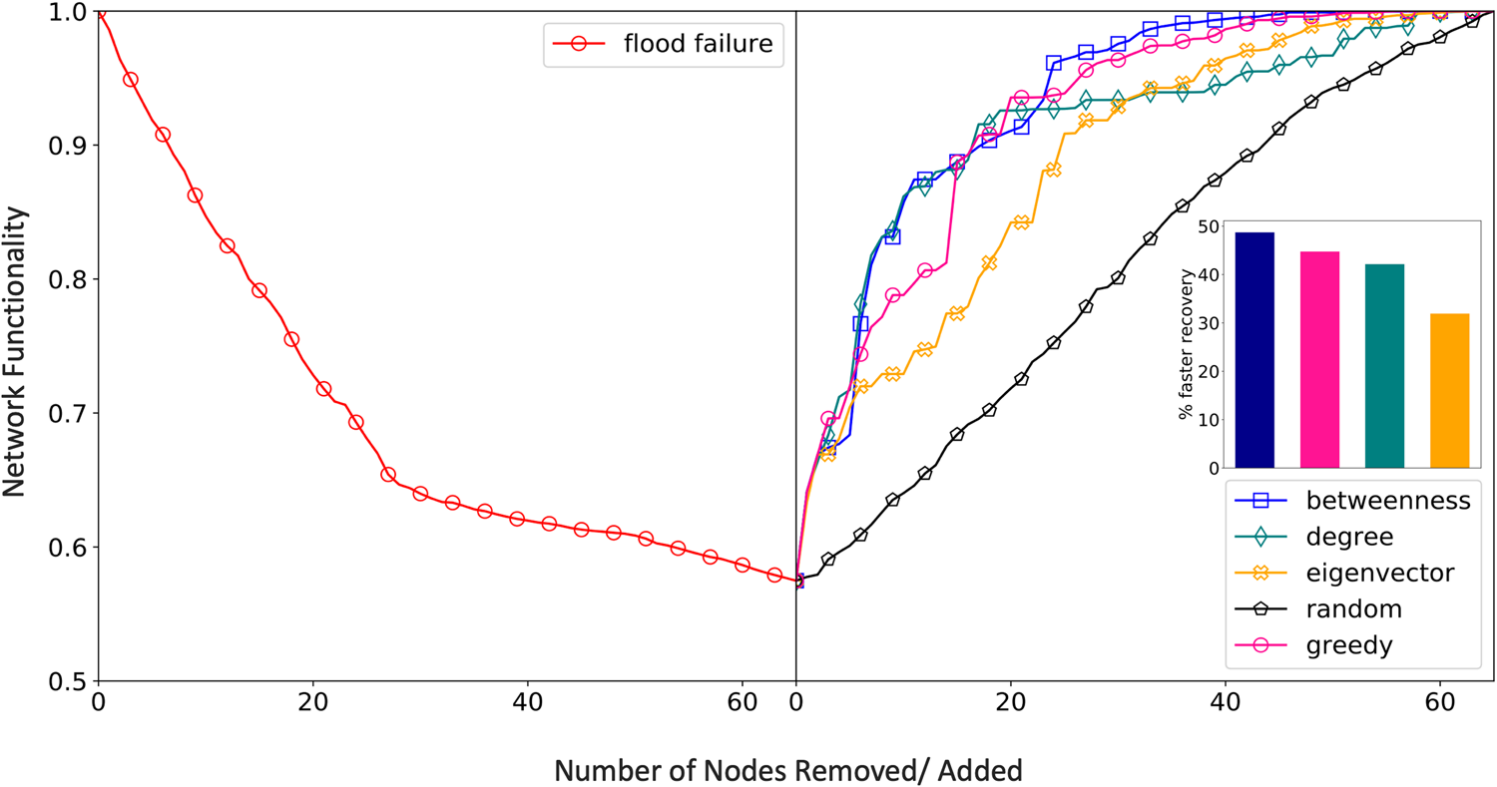
Post-Failure Recovery. Recovery strategies for restoration of the London Rail Network. Three network centrality-based and one optimization-based (greedy) recovery sequences are simulated and compared. Random recovery is plotted as baseline. Efficiency of each approach is measured by the area between the curve and y-axis. Lower area equates to faster recovery. Recovery is fastest when nodes are added in order of their betweenness values.

In the absence of real data on traffic flow and restoration cost per station, we make reasonable assumptions to perform our analysis: (1) restoration cost per node is proportional to its degree (2) betweenness values for each node have been used as a proxy for network flow in prior literature^37^ and thus, it is used to represent node weights (traffic flow). Figure 4 compares the recovery strategies based on different centrality measures and optimization-based greedy algorithm (GA) [see Methods]. Random recovery is plotted as a baseline.

We observe that network-centrality based recovery approaches perform comparably to the optimization-based GA approach with one scenario (recovery based on node betweenness) outperforming GA approach by ~7%. Greedy algorithms which make optimal decisions at each step (by maximizing functionality gain) do not guarantee a global optimum as evident in this case. For example, some nodes which may not have the highest functionality gain have to be recovered first to achieve faster recovery in later steps.

In transportation networks where real-world node importance is highly correlated with node centrality, centrality-based recovery methods offer a computationally cheap and intuitive approach for efficient network recovery. While a purely centrality-based recovery order is agnostic to specific restoration demands and goals, here we show, how problem specific constraints such as restoration cost can easily be incorporated into a topology-driven network functionality metric to achieve comparable performance to more rigorous network optimization techniques. Infrastructure owners and operators may have direct access to data about resources, constraints, and system vulnerabilities which could augment the methodologies proposed and demonstrated here. While the analysis points to an organizing principle where a quick near-optimal recovery sequence could be generated using centrality-based measures, there is a need to further test the hypothesis on other multimodal transportation NoNs.

### Realistic scenario – flooding of the underground network

Taking a cue from a real incident in 2012 when Hurricane Sandy inundated a major section of the New York subway (e.g., see Fig. 2 in^38^), we model a similar disruption in the LRN. Here, we remove the flooded nodes only from the Underground layer. Due to inter-network dependency, failure will be propagated to the other two layers.

**Figure 5:**
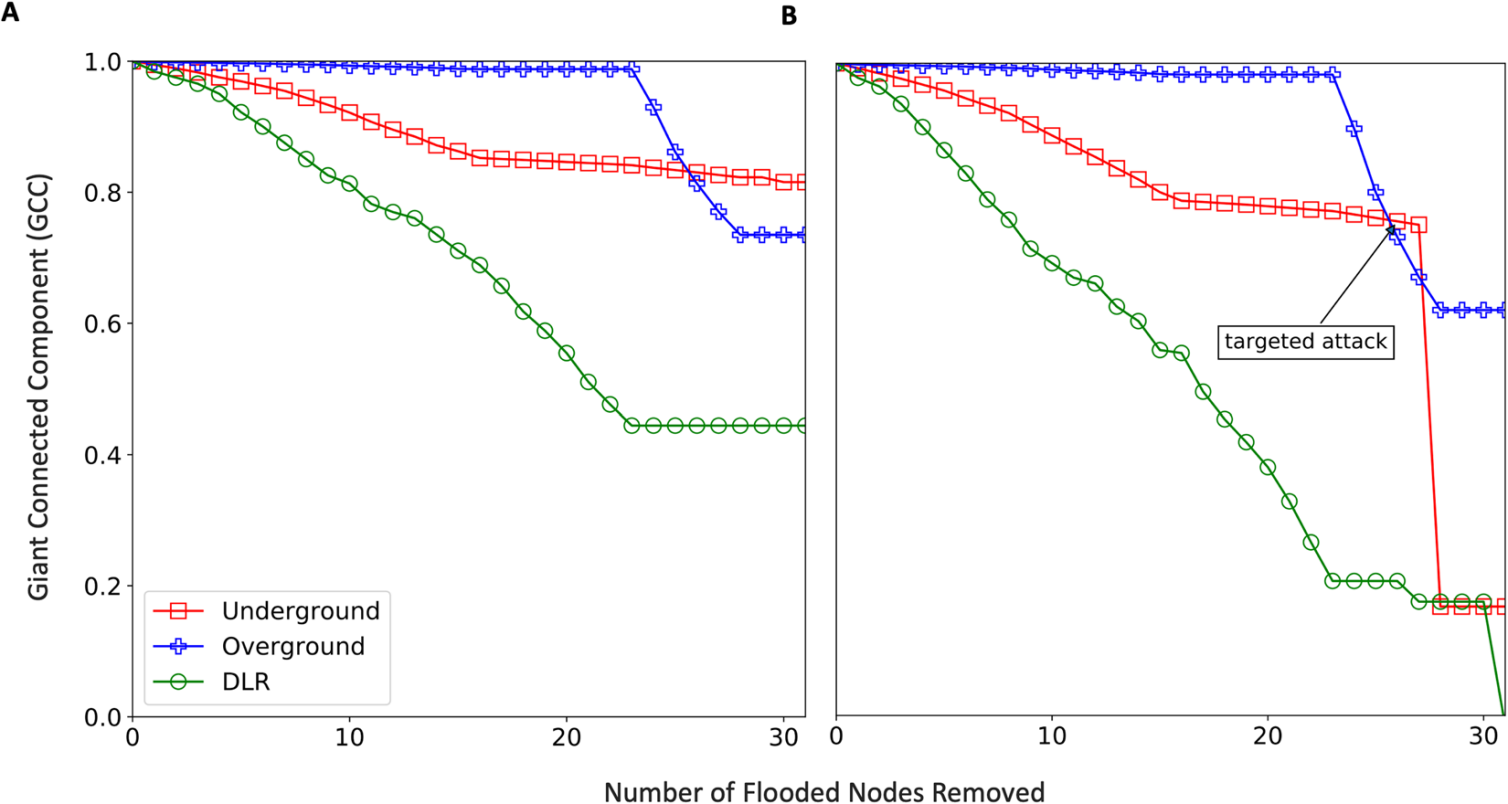
Indirect Flood Failures due to Interdependency. Flood failure is restricted to the underground network and indirect failure in the other two networks is analyzed. **(A)** Flood-only failure **(B)** Compound failure – same as before. As observed, indirect failure can be disproportionate due to different criticality of the same node across networks.

**Figure 6:**
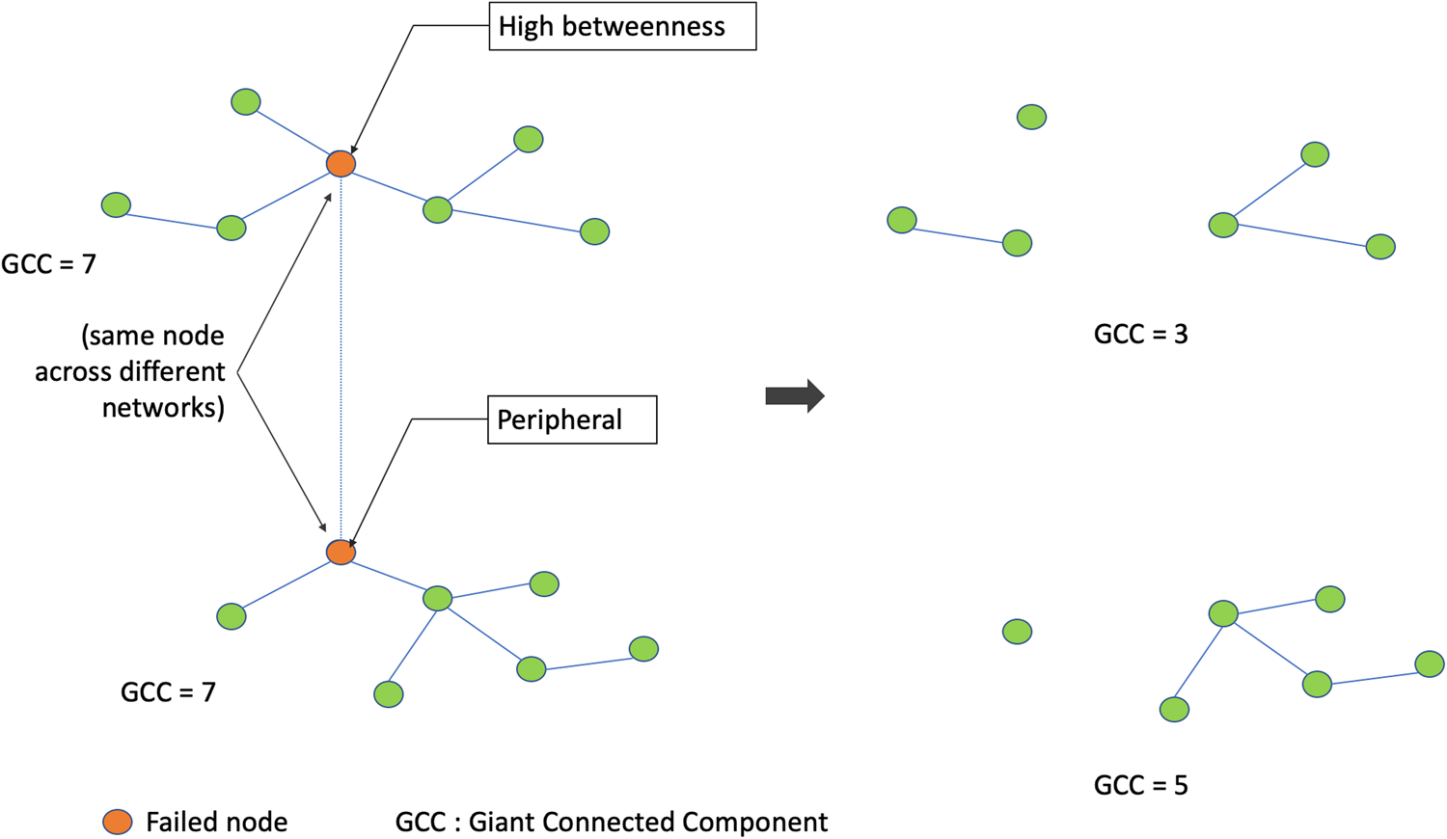
Node Criticality and Severity of Indirect Failure. Schematic figure to demonstrate extent of indirect failure given failed node with different criticality (e.g. betweenness) across interdependent network. Node in orange is the failing node and GCC is size of the giant (largest) connected component. A higher node criticality translates to greater loss of network functionality (lower size of final GCC).

Figure 5 shows how indirect failures caused by cascading failures can be disproportionately severe in the dependent networks, as exemplified here in the LRN. This is because the same lower degree nodes in one network can be more critical (with higher degree) in the dependent networks and cause greater disruption, an effect that may multiply when multiple such nodes and networks are considered. Figure 6 presents a schematic illustration of this process. For completeness, a compound-failure case is simulated as well (Fig. 5(B)). We observe that a 5% targeted attack can amplify the network damage by multiple times (over 300% in the case of LRN Underground) and may even cause a complete failure in a dependent network, such as the DLR in this case (Fig. 5(B)).

## Discussion

As urbanization and climate change intensifies, understanding and improving the resilience of urban lifeline infrastructure is critical. Here we have presented an end-to-end assessment framework to quantify the resilience of multimodal urban transport systems (MUTS) modeled as network-of-networks (NoNs). We address three hypotheses focusing on the (1) spatial constraints of real-world infrastructure networks, (2) the growing threat of compound disruptions and (3) network-science based post-disruption recovery principles. The corresponding insights are summarized in Table 3.

**Table 3:** Resilience Framework for London Rail Network-of-Network.

Insights Summary:
London Rail NoN is more vulnerable compared to its spatially non-embedded but topologically equivalent NoNs.
Compound disruptions cause network-wide failure but it is not disproportionate.
Centrality-based methods outperforms optimization-based greedy algorithm for post-disruption optimal recovery in LRN

Based on our findings here and insights in the literature^39,40^, we suggest that urban transportation systems, being a type of spatially embedded networks, have intrinsic topological vulnerabilities that are considerably different from their non-spatial counterparts. We need to understand how these spatial constraints, such as a short characteristic link length, affects the robustness properties of these systems. Thus, theoretical frameworks (such as^17,21–23^) in the extant network science literature which may have been developed for non-spatial networks, may not directly translate to the real-world without incorporating the spatial nature of these infrastructure networks. This finding points to the need for broadening network science studies in the context of real-world infrastructure resilience by developing theoretical and empirical studies, as well as methods and tools, that are geared toward spatially embedded networks.

A second advancement vis-à-vis^11,20^ is that we consider the emerging threat of compound disruptions, especially targeted attacks in conjunction with natural hazards. We examined whether compound failures can potentially cause disproportionate damages when network capacity is already weakened. We find that compound disruptions indeed cause network-wide failures; however, a major proportion of the loss of functionality is still contributed by targeted attacks, even in the limiting case of 5% targeted attack. Under the assumptions considered, a disproportionate compound failure impact is not observed, irrespective of whether targeted attacks occur before or after the natural hazard. While the result may seem intuitive, an interesting hypothesis to pursue would be if the adversary can design ingenious targeted attacks (not just based on node centralities) in a manner so as to exacerbate the impact of impending natural disaster, for e.g. removing specific nodes which may proliferate flooding across the network. In such a scenario, a truly compound failure may be observed. Our work is an early exploration of networked systems under compound attacks and a hypothesis-driven study which explores this topic in more depth both from the perspective of network science theory and engineering practice appears to be a clear and present need.

Furthermore, we examine the viability of network centrality-based recovery methods vis-à-vis optimization-based methods and find the former to outperform for MUTS such as the LRN. While generalization would require testing on varied datasets, the results point to an exciting direction where centrality-based heuristic recovery can offer viable alternatives to network optimization methods, especially when system related granular information for developing accurate objective functions is hard to come by. In addition to that, with a novel network functionality metric, we showcase how problem specific constraints can be baked into it to derive recovery sequences which are more than just topology driven.

Urban rail systems in long-established megacities (especially in the developed nations) such as London or New York are more than a century old when the idea of system-level resilience may not have been the highest priority. Our results here show how design decisions which maximized efficiency and relatively obsolete considerations (e.g., the need for major stations to be near water bodies) from pre-modern times rendered the London Rail Network highly vulnerable to flood-induced failure. Thus, there is a need for designers of new urban lifeline infrastructure networks (including transportation), as well as for owners and operators of existing urban lifeline systems, to embed resilience considerations and move beyond traditional design practices which focus only on maximizing efficiency and structural longevity. This is especially true under growing threats of urban floods and other natural hazards, as well as compound disruptions such as opportunistic cyber or cyber-physical attacks. Future research needs to strengthen these insights by considering multiple urban systems across geographies and lifeline sectors.

Our findings and frameworks lead to potentially new hypotheses and research directions, especially for real-world interdependent spatial networks, some of which have been described earlier in this section. As a next step, there is a need to incorporate system dynamics and flow to simulate more realistic operational conditions, such as the case partially functional nodes. In addition, the giant component size (GCC) as a robustness measure may be more suited for non-spatial networks, thus there is a need to explore alternative metrics which consider network dynamics. Finally, considering multiple failure schemes (including but not limited to cascading failure schemes considered here) and contingency analysis of networked systems (including unknown or potentially unknowable threats) may yield insights of value to both researchers and practitioners.

## Methods

### Dataset availability

The 2013 London metro dataset used in the paper has been made available by De Domenico *et al*.^41^. The dataset has been cited with permission from Transport for London (https://tfl.gov.uk/). The flood risk map is obtained from the UK’s Environment Agency database^35^. Python codes corresponding to this paper are freely available online on Github: https://github.com/nisyad/LRN_NoN_Resilience

#### Network structure

**Table:** 

Network Layer	Nodes (Stations)	Links
Underground	271	312
Overground	83	83
DLR	45	46

### Common stations

There are 24 stations common between the Underground and Overground networks, 5 stations common between the Underground and DLR, and 1 station is common between the Overground and DLR.

### Failure scenarios

#### Flood failure

The flooded nodes are divided into three categories based on their proximity to river Thames. Considering the outward spread of the flood, the first category of nodes closest to the river fail first and so on. To account for uncertainty, nodes within each category may fail randomly. 20 independent runs are considered to model flood failure.

#### Random failure

Nodes are removed randomly from the overall network.

#### Random-local failure

Using the lat-lon values of station locations, a distance matrix is generated for the entire network where each entry (i, j) is the distance between station i and j. 100 local clusters of geographically closest 65 nodes (same number as flooded nodes) are generated across the network to model a localized failure. Within each cluster, nodes fail at random. 20 independent runs considered.

#### Targeted failure

A predetermined order of nodes based on some metric of importance is considered and nodes are removed in the decreasing order of importance. Here the importance metric used are node degree and betweenness centrality.

#### Compound failure

After a certain level of flood failure has occurred, a targeted attack is introduced by removing n% of nodes based on degree centrality. The overall number of failed nodes are kept equal to the number of total flooded nodes.

### Recovery methods

#### Centrality-based Recovery

Nodes are added back to the network in decreasing order of the chosen node centrality metric, for e.g., betweenness. In other words, nodes with high betweenness are added first.

#### Greedy algorithm

The recovery sequence is identified by making locally optimal choices. The objective function describing the network functionality is maximized and at each recovery step, the nodes which restores maximum capacity is added back to the network.
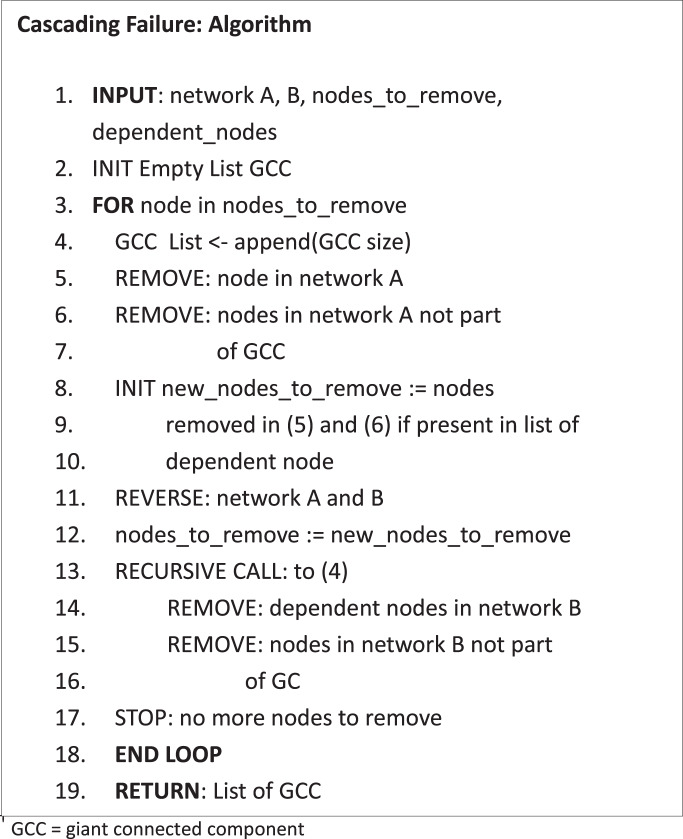

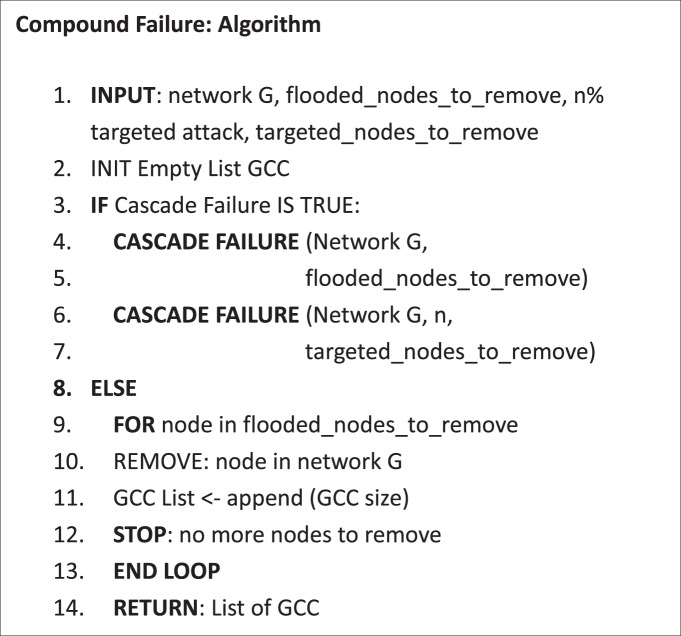


### Centrality measures

Complex network structures are very heterogenous and some nodes are expected to be more important than other nodes which can be quantified by node centrality measures.

#### Degree centrality

Number of nodes linked to a particular node.

#### Betweenness centrality

For every pair of nodes, there exists a shortest path. The betweenness centrality of a node is number of such of shortest paths passing through it.

$$g(v)=\sum _{s\ne v\ne t}\frac{{\sigma }_{st}(v)}{{\sigma }_{st}}$$

${\sigma }_{st}$ is the total number of shortest paths from node $s$ to $t$ and ${\sigma }_{st}(v)$ is the total number of shortest paths passing through node $v$.

#### Eigenvector centrality

A node can be considered important if it is connected to other important nodes. This importance of node $i$ can be quantified by a vector ${x}_{i}$:

$${x}_{i}=\frac{1}{\lambda }\mathop{\sum }\limits_{k=1}^{N}{A}_{k,i}{x}_{k}$$

where, $\lambda$ is a non-zero constant. In the matrix form:

$$\lambda x=Ax$$

The importance of node $i$ is defined by the left-hand eigenvector of the adjacency matrix $A$ associated with eigenvalue $\lambda$. The entries of $x$ are called eigenvector centrality.

## Supplementary information

Supplementary information.
