# Supplementary material for: Resilience of Urban Transport Network-of-Networks under Intense Flood Hazards Exacerbated by Targeted Attacks
Source: Sci Rep. 2020 Jun 25;10:10350. doi: 10.1038/s41598-020-66049-y (PMC7316753; doi:10.1038/s41598-020-66049-y)

**SUPPLEMENTARY INFORMATION**

**Resilience of Urban Transport Network-of-Networks under Intense Flood Hazards Exacerbated by Targeted Attacks**

**Nishant Yadav**^1^, **Samrat Chatterjee**^2,†^, **Auroop R. Ganguly**^1,†^

^1^Sustainability and Data Sciences Laboratory, Department of Civil and Environmental

Engineering, Northeastern University, Boston, MA, USA

^2^Computing and Analytics Division, National Security Directorate, Pacific Northwest National

Laboratory, Richland, WA, USA

^†^Corresponding Authors: a.ganguly@northeastern.edu; samrat.chatterjee@pnnl.gov

**Supplementary Figures**

**Figure S7** | Schematic Cascade Failure in Partially Interdependent Network. In stage I, a node is removed due to direct failure (orange color). In stage II, all the dependent nodes in both layers are removed (yellow color). In stage III, dependent nodes on the removed node in the other layer are removed. Stage IV represents a feedback mechanism where dependent nodes (in first layer) on the failed nodes in the other layer are also removed (purple color). Green nodes represent functional nodes in each stage as part of the giant connected component.

**
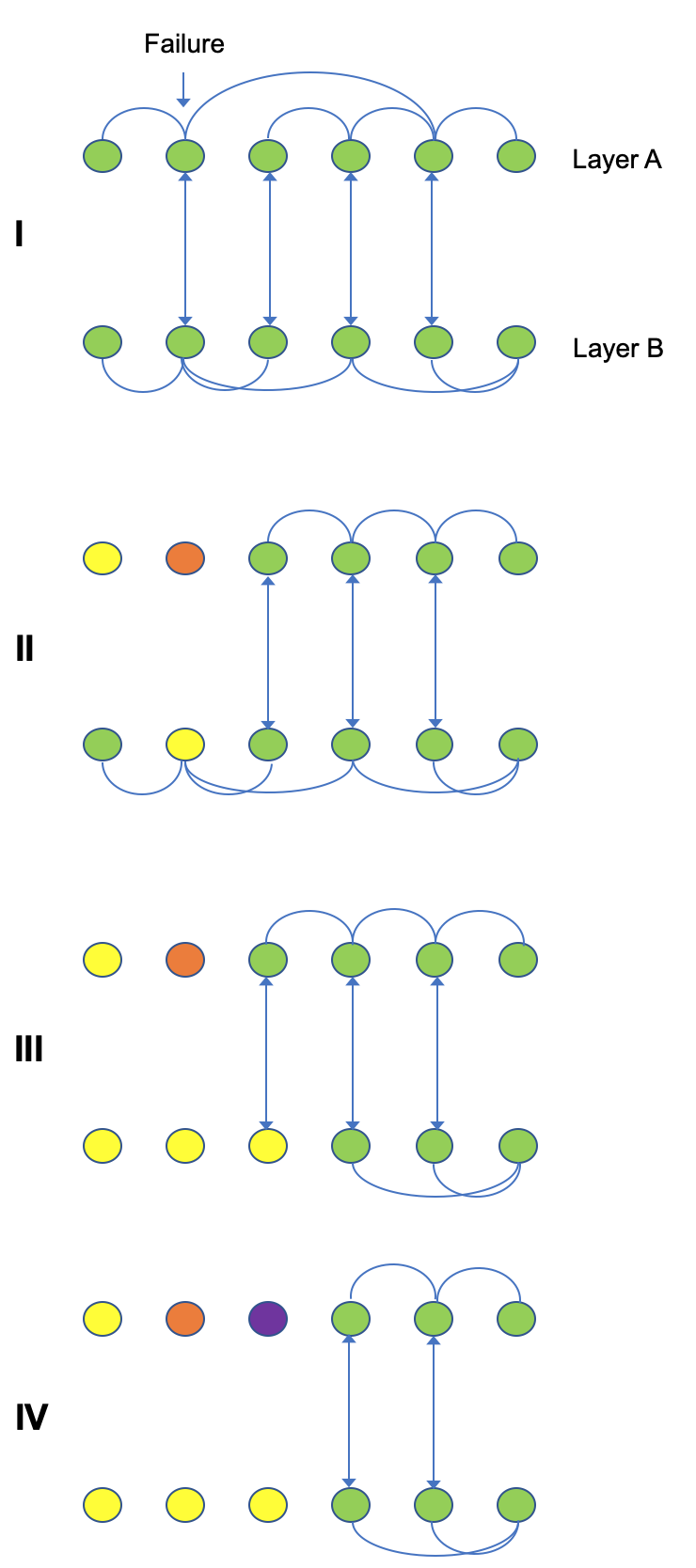
**

**Figure S8** | Degree Distribution of the London Rail Network. Equivalent Erdos-Renyi and Scale-Free (SF) network degree distributions are plotted for comparison. Degree-distribution of the scale free network is curtailed at 14 in the plot.


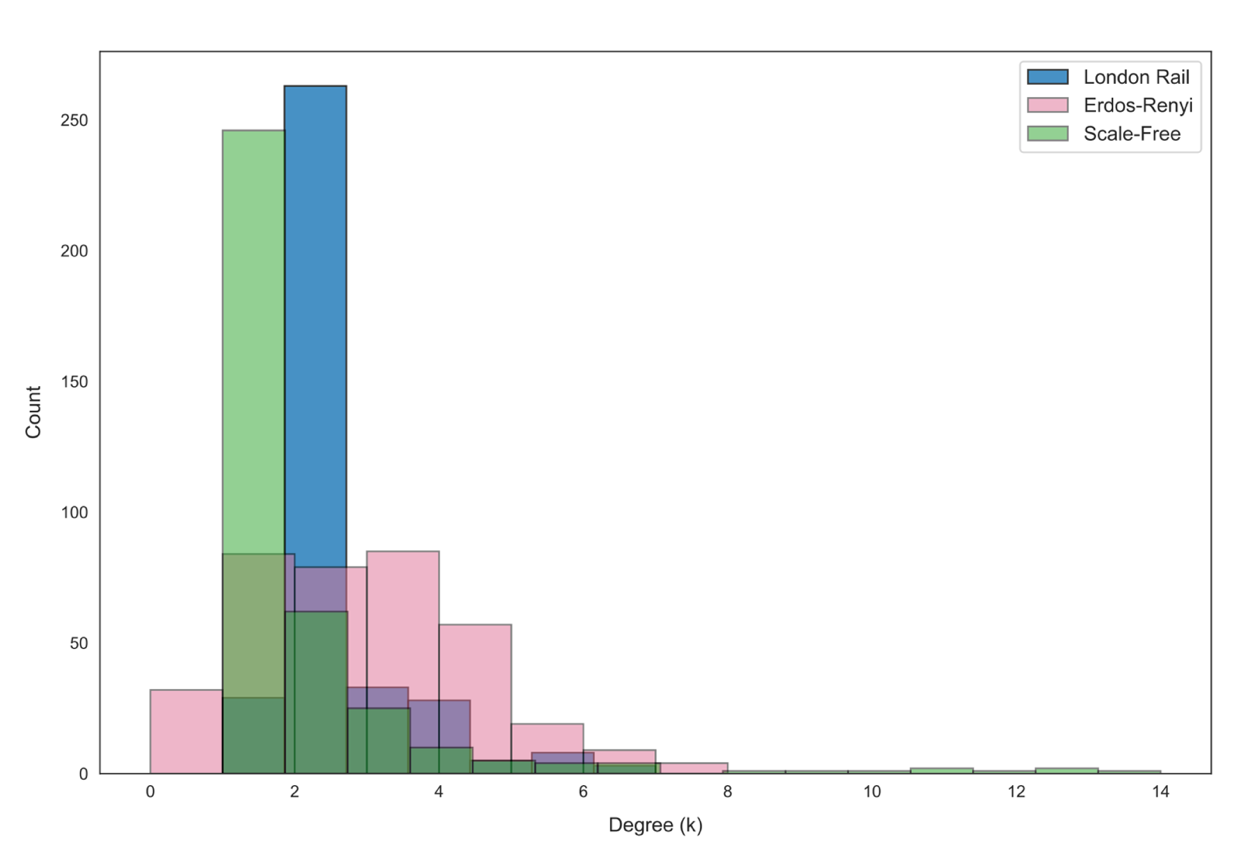


**d**

**Figure S9** | Degree Distribution of the Flooded Network vs the Total Network. Higher average degree for the flooded network indicates the presence of more critical nodes in the flooded area.


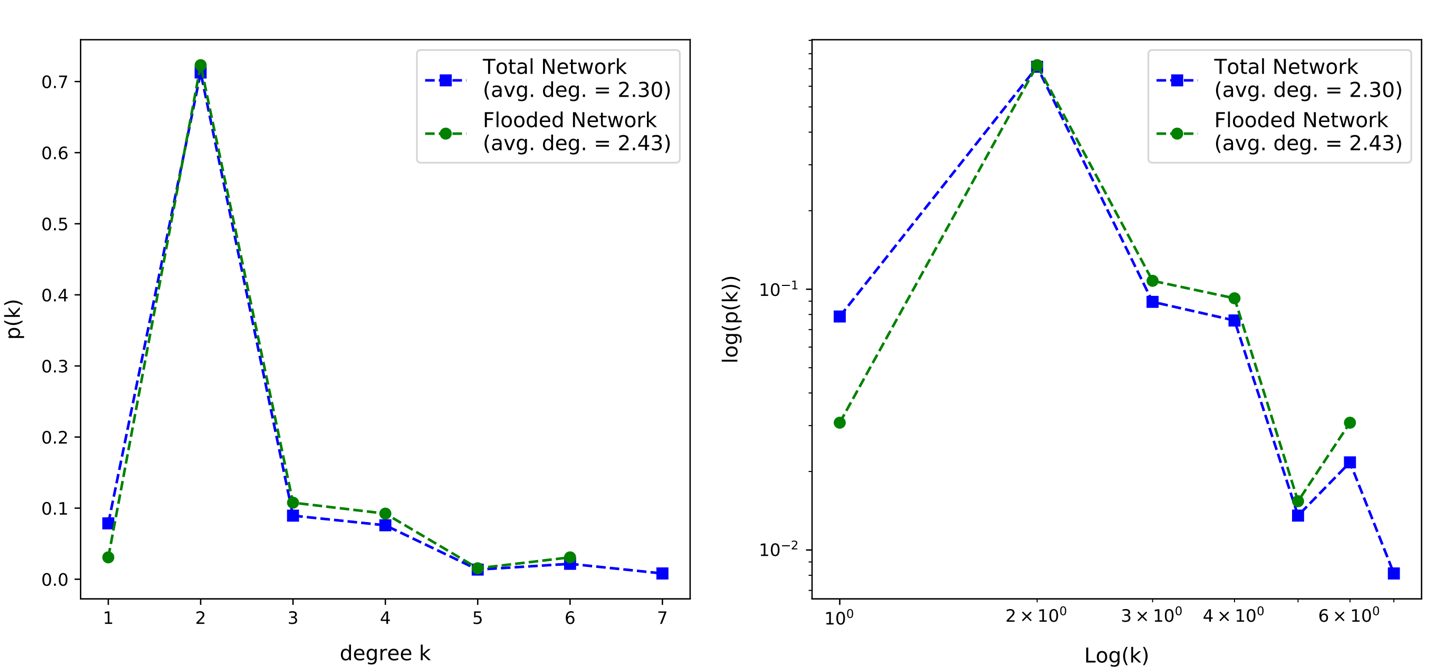

Supplement: Supplementary file 1 — Supplementary information. [file 41598_2020_66049_MOESM1_ESM.docx]
